# Application of Response Surface Methodology for Optimizing Arginine Deiminase Production Medium for *Enterococcus faecium* sp. GR7

**DOI:** 10.1155/2013/892587

**Published:** 2013-12-17

**Authors:** Baljinder Kaur, Rajinder Kaur

**Affiliations:** Department of Biotechnology, Punjabi University, Patiala 147002, Punjab, India

## Abstract

Arginine metabolism in *Enterococcus faecium* sp. GR7 was enhanced via arginine deiminase pathway. Process parameters including fermentation media and environmental conditions were optimized using independent experiments and response surface methodology (central composite design). Fermentation media (EAPM) were optimized using independent experiments which resulted in 4-fold increase in arginine deiminase specific activity as compared to basal medium. To further enhance arginine deiminase activity in *E. faecium* sp. GR7 and biomass production including a five-level central composite design (CCD) was employed to study the interactive effect of three-process variables. Response surface methodology suggested a quadratic model which was further validated experimentally where it showed approximately 15-fold increase in arginine metabolism (in terms of arginine deiminase specific activity) over basal medium. By solving the regression equation and analyzing the response surface cartons, optimal concentrations of the media components (g/L) were determined as arginine 20.0; tryptone 15.0; lactose 10.0; K_2_HPO_4_ 3.0; NaCl 1.0, MnSO_4_ 0.6 mM; Tween 80 1%; pH 6.0 for achieving specific arginine deiminase activity of 4.6 IU/mG with concomitant biomass production of 12.1 mg/L. The model is significant as the coefficient of determination (*R* ^2^) was 0.87 to 0.90 for all responses. Enhanced arginine deiminase yield from *E. faecium*, a GRAS lactic acid bacterial strain, is desirable to explore *in vitro* therapeutic potential of the arginine metabolizing *E. faecium* sp. GR7.

## 1. Introduction 

**Figure 1:**
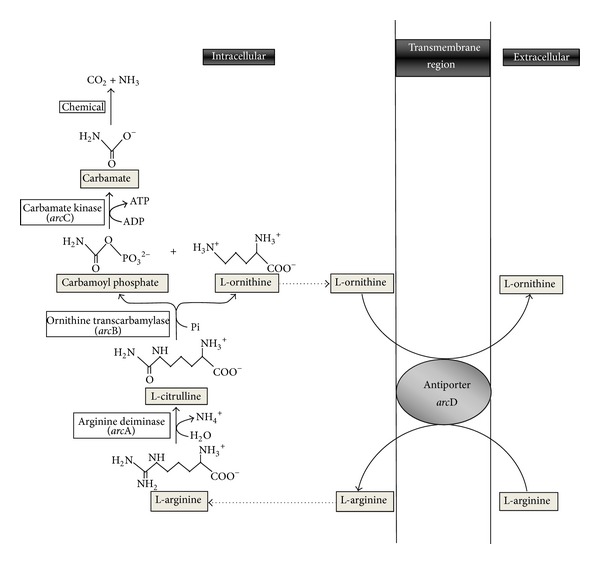
Arginine catabolism in *E. faecium. *

L-arginine is classified as conditionally essential amino acid for protein synthesis that is metabolized through citrulline, ornithine, creatine, proline, and polyamines in human body [1, 2]. It is used by a number of microorganisms to generate ATP fermentatively via arginine deiminase (ADI) pathway which is also known as arginine dihydrolase (ADH) pathway [3]. Arginine is metabolized by three successive enzymatic reactions (Figure 1), involving arginine deiminase (ADI; EC 3.5.3.6) to citrulline and ammonia, ornithine transcarbamylase (EC 2.1.3.3) to ornithine and carbamoyl phosphate and finally to ATP by carbamate kinase (EC 2.7.2.2) [4]. ADI activity is previously reported in many lactic acid bacteria including *Enterococcus faecalis, Lactobacillus sp., Lactococcus, Leuconostoc, Oenococcus, Streptococcus*, and *Weissella* [3, 5–10]. In general, arginine induces expression of ADI pathway enzymes, and some carbohydrates such as glucose and galactose are known to control their synthesis by catabolite repression [9].

The present study was aimed to screen and optimize media components that increase ADI activity of *Enterococcus* sp. RSM, formerly known as Box-Wilson methodology, is the most widely used statistical technique to evaluate relationship between a set of controllable experimental factors and observed results [11]. RSM is capable to find optimum set of experimental factors that produce maximum or minimum value of response and represent the direct and interactive effect of process variables through two-dimensional and three-dimensional graphs. Keeping in view, the great commercial application of ADI as a therapeutic agent; an attempt was made to optimize the composition of the ADI production media for *E. faecium* sp. GR7 which was carried out in two steps. Independent experiments for the selection of most influential process parameters including fermentation media and environmental conditions were performed followed by application of CCD design for final optimization of media components to enhance ADI activity and biomass production using influential process variables.

## 2. Materials and Methods

### 2.1. Bacterial Strain and Culture Medium Used

*E. faecium* sp. GR7 used in the study was cultured in MAM medium (g/L) consisting of tryptone 10.0; glucose 5.0; yeast extract 5.0; arginine 3.0; KH_2_PO_4_ 0.5; MgSO_4_ 0.2; MnSO_4_ 0.05; Tween 80 1.0 mL/L; Agar 2.0; pH 6.0 [12]. Optical density of the inoculums was adjusted to 1.0, and 1% v/v culture was used in each experiment. Cultures were incubated at 37°C for 24 h.

### 2.2. Enzyme Assay

To determine enzyme activity, 24 h-old cultures were centrifuged at 8,000 rpm for 10 min. Cell free supernatant (CFS) was assessed for extracellular protein and enzyme activity. For assaying intracellular ADI activity, cell pellet was resuspended in lysis buffer. Total protein was estimated by measuring absorption at 280 nm using standard curve of BSA. ADI activity was assayed using standard method of De Angelis et al. [12]. Assay mixture consisted of 150 *μ*L of 50 mM arginine, 2.3 mL of 50 mM acetate buffer (pH 5.5), 50 *μ*L of cell wall or cytoplasm preparation, and 3.6 *μ*L of sodium azide (0.05% w/v). Controls without substrate and without enzyme were included. After incubation at 37°C for 1 h, the reaction was stopped by adding 0.5 mL solution of 2N HCl, and precipitated protein was removed by centrifugation. Citrulline content after enzyme assay in CFS was determined by Archibald's method [13]. One milliliter of the supernatant was added to 1.5 mL of an acid mixture of H_3_PO_4_-H_2_SO_4_ (3/1 v/v) and 250 *μ*L of diacetyl monoxime (1.5% 2, 3 butanedione monoxime) in 10% (v/v) methanol, mixed and boiled in the dark for 30 min. After cooling for 10 min, absorbance was measured at 460 nm and one enzyme unit was calculated as the amount of enzyme required to catalyze formation of 1 *μ*mol citrulline per min. Finally, specific activity was calculated as international enzyme units present per mg (IU/mG) of protein.

### 2.3. Selection of Basal Medium for ADI Production

Six culture media including MAM [12]; MRS [14]; TGYE [15]; (modified) MRS broth [12]; *Enterococcus* confirmatory broth [16]; and M9 Minimal salt media [17] each supplemented with arginine (15 mM) were selected to investigate production of ADI in *E. faecium* sp. GR7. Inoculums were subcultured twice in MAM broth at 37°C for 24 h and used at 1% v/v for each experiment. ADI specific activity was determined following standard procedures of De Angelis et al. [12] and Archibald [13].

### 2.4. Optimization of Process Parameters for Enhancing ADI Activity

*E. faecium* sp. GR7 was grown in the selected medium (TGYE) and the effect of various parameters, that is, initial pH (5.0 to 7.0), inoculum size (1 to 5% v/v), subculturing period (1 to 4 days), incubation temperature (25°C to 50°C), and culture incubation conditions, that is, aerobic, anaerobic, shaking (200 rpm) on ADI production was studied individually, by varying one factor at a time. At each step, the selected factor was included in the basal medium (selected from the previous experiment) for getting a set of conditions that enhanced ADI activity in *E. faecium* sp. GR7. Rest of the conditions and enzyme assays were described previously.

### 2.5. Effect of Various Nutrients on Enzyme Production

The effect of various carbon and nitrogen sources on enzyme production in *E. faecium* sp. GR7 was investigated. In the production medium, carbon of the basal media was replaced with glucose, galactose, sucrose, maltose, lactose, and fructose, and nitrogen with yeast extract, peptone, tryptone, and beef extract which were tested at levels ranging from 5 to 20 g/L (w/v).

### 2.6. Effect of Inducer Concentration

Effect of inducer concentration, that is, arginine on ADI production was studied by supplementing basal medium with different concentrations of arginine from 5 to 20 mM (w/v).

### 2.7. Effect of Salts and Surfactants on Enzyme Production

Basal medium was supplemented with various salts such as NaCl (0.5–5 g/L) (w/v), K_2_HPO_4_ (0.5–5 g/L) (w/v), MnSO_4_ (0.2–2 mM) (w/v), CuSO_4_ (0.025–0.075 mM) (w/v), ZnSO_4_ (2–6 mM) (w/v), and surfactants including CTAB, SDS, Tween 80, and Triton X-100 (0.1% w/v) to investigate their role in improving enzyme production and secretion by *E. faecium* sp. GR7.

### 2.8. Experimental Design

Important process variables, identified based on independent experiments, were finally used to optimize composition of the ADI production medium for *E. faecium* sp. GR7. RSM is a successive and exploratory tool for establishing the influence and interaction among variables on biological activities [18]. Experimental design central composite design (CCD) of RSM using Design Expert Software trial version 8.0.2 statistical software (State-Ease Inc., Minneaopolis, MN, USA) was applied for improving enzyme activity and cell densities in the LAB isolate, that is, *E. faecium* sp. GR7. A quadratic model obtained by a multiple regression technique for three factors, that is, tryptone, lactose, and arginine was studied at five different levels along with four constant variables, so that, interactions among these variables at different levels could be studied for two responses, that is, ADI activity and biomass (Table 1). During CCD experiments, concentrations of K_2_HPO_4_ 3 g/L, NaCl 1 g/L, MnSO_4_ 0.6 mM, and Tween-80 1%, pH 6.0, and temperature 30°C for 24 h under aerobic conditions were kept as constant factors. In all the CCD experimental runs, biomass and enzyme activities were assayed using standard protocols as described previously. A total of 20 experiments were employed in CCD to estimate curvature and interaction effects of selected variables, and finally, significance of the obtained model was checked by *F*-test and goodness of fit by multiple correlation *R* as well as determination *R* ^2^ coefficients. All design matrices were generated and analysed using Design-Expert 8.0.2 to illustrate the relationships between experimental and predicted values, and the results were depicted as 2D contour plots.

### 2.9. Experimental Validation of Statistical Model

The response surface model was validated under the predicted conditions in triplicates. On the basis of results obtained in statistical RSM analysis, the optimized medium (g/L) was composed of tryptone 15.0; lactose 10.0; arginine 20.0; MnSO_4_ 0.6 mM; NaCl 1.0; K_2_HPO_4_ 3.0; Tween 80 1%; pH-6.0. The media were inoculated with *E. faecium* sp. GR7 (1% v/v) and incubated at 30°C for 24 h. The samples were collected after 4 h intervals to estimate specific ADI activity and growth *A*_600_ for optimized and unoptimized media basal medium.

### 2.10. Statistical Analysis

One way Anova analysis was carried out, and the results are presented as mean ± standard deviation of three triplicate experiments. A probability value of *P* value <0.05 was used as the criterion for statistical significance.

## 3. Results and Discussion

### 3.1. Selection of Basal Medium for ADI Production

**Figure 2:**
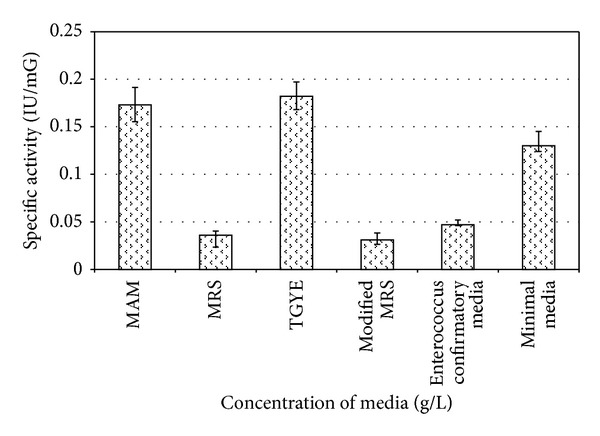
Effect of culture media on specific ADI activity of *E. faecium* sp. GR7. Results are expressed as the mean ± S.D (*n* = 3), *P* value <0.05.

MAM, MRS, TGYE, MRS (modified), *Enterococcus* confirmatory media, and M9 minimal salt media were selected on the basis of previous literature citations for studying ADI enzyme production in *E. faecium* sp. GR7. The highest specific ADI activity of 0.182 ± 0.001 IU/mG was observed in TGYE media when supplemented with 15 mM arginine (as shown in Figure 2), which was selected as a basal media for further study of process parameter variables on nutrients, inducers, salts, surfactants, and processing parameters on ADI production of *E. faecium* sp. GR7.

### 3.2. Effect of Initial pH, Temperature, and Culture Conditions on Arginine Production

**Figure 3:**
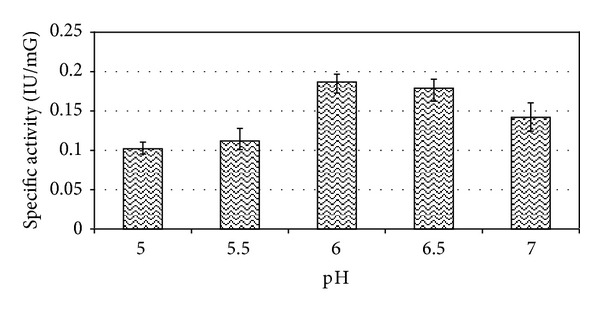
Effect of pH on specific ADI activity of *E. faecium* sp. GR7. Results are expressed as the mean ± S.D (*n* = 3), *P* value <0.05.

**Figure 4:**
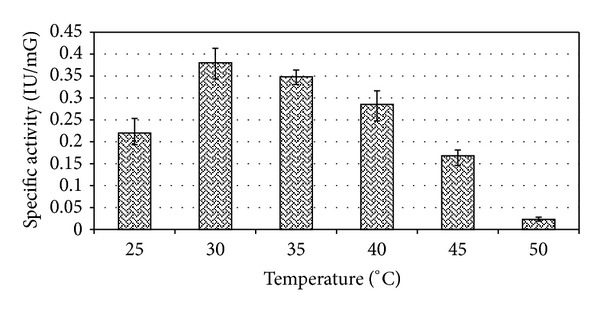
Effect of temperature on ADI enzyme production in *E. faecium* sp. GR7. Results are expressed as the mean ± S.D (*n* = 3), *P* value <0.05.

**Figure 5:**
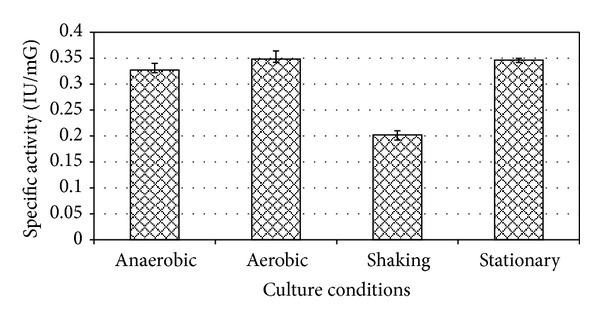
Effect of culture conditions on enzyme production. Results are expressed as the mean ± S.D (*n* = 3), *P* value <0.05.

When grown in basal media *E. faecium* sp. GR7 yielded maximum ADI activity of 0.187 IU/mG at pH 6.0 when incubated at 30°C for 24 h (Figures 3 and 4), which was better when the cultures were kept under aerobic and stationary conditions (Figure 5).

### 3.3. Effect of Inoculum and Subculturing

**Figure 6:**
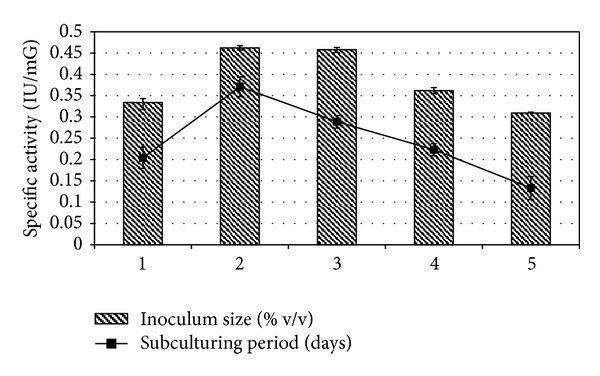
Effect of inoculum size and subculturing period on enzyme production. Results are expressed as the mean ± S.D (*n* = 3), *P*-value <0.05.

Maximum ADI production in *E. faecium* sp. GR7 was reported with 2% (0.462 IU/mG) inoculum size and after 2nd subculturing period (Figure 6).

### 3.4. Effect of Carbon and Nitrogen Sources

**Figure 7:**
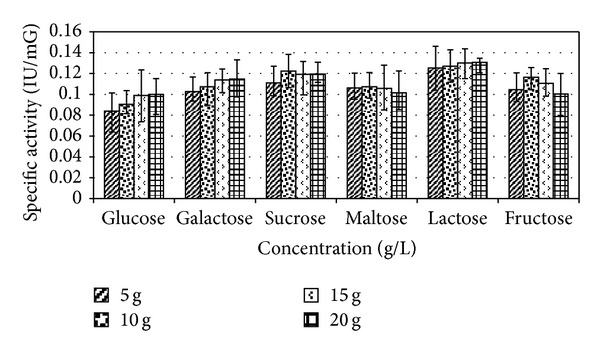
Effect of sugars on enzyme production. Results are expressed as the mean ± S.D (*n* = 3), *P*-value <0.05.

**Figure 8:**
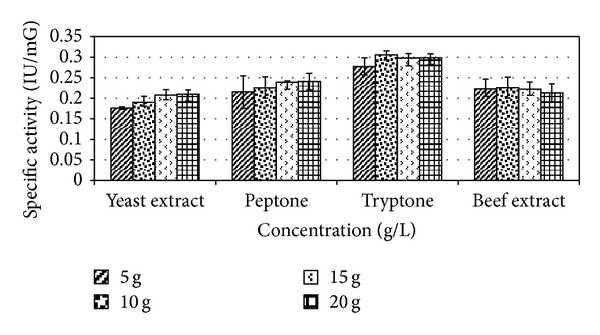
Effect of different nitrogen concentrations on enzyme production. Results are expressed as the mean ± S.D (*n* = 3), *P*-value <0.05.

Among the carbon sources, lactose supported a maximum specific ADI activity at concentration (20 g/L) of 0.131 IU/mG (Figure 7). Tryptone gave maximum enzyme activity of 0.306 IU/mG at concentration of 10 g/L (Figure 8).

### 3.5. Effect of Arginine

**Figure 9:**
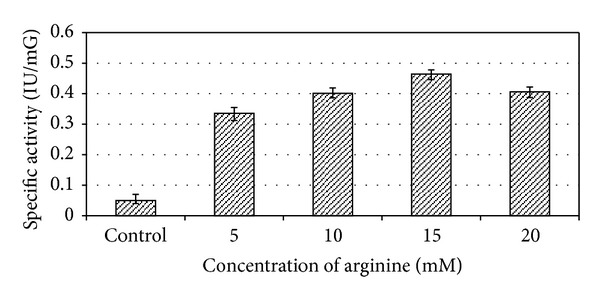
Effect of arginine (inducer) concentration on specific ADI activity of *E. faecium* sp. GR7. Results are expressed as the mean ± S.D (*n* = 3), *P*-value <0.05.

The ADI pathway is an arginine inducible system in most of the microorganisms [5, 9, 12, 25, 27–29]. In our study, maximum specific ADI activity of 0.464 ± 0.014 IU/mG for *E. faecium* sp. GR7 was obtained in production media supplemented 15 mM arginine as compared to unsupplemented basal media as shown in Figure 9.

### 3.6. Effect of Salt and Surfactants

**Figure 10:**
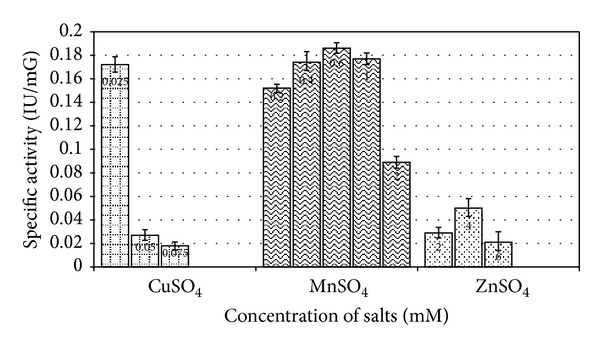
Effect of various metal ions on specific ADI activity. Results are expressed as the mean ± S.D (*n* = 3), *P*-value <0.05.

**Figure 11:**
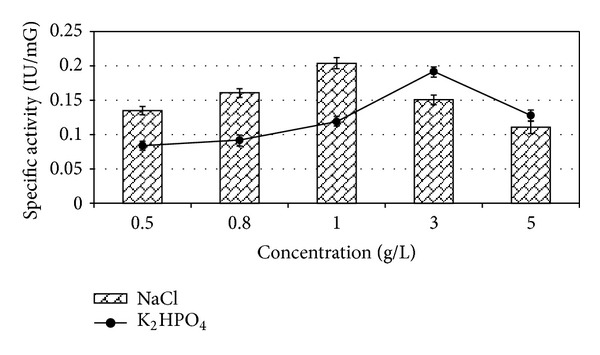
Effect of salt concentrations on specific ADI activity by *E. faecium* sp. GR7. Results are expressed as the mean ± S.D (*n* = 3), *P*-value <0.05.

**Figure 12:**
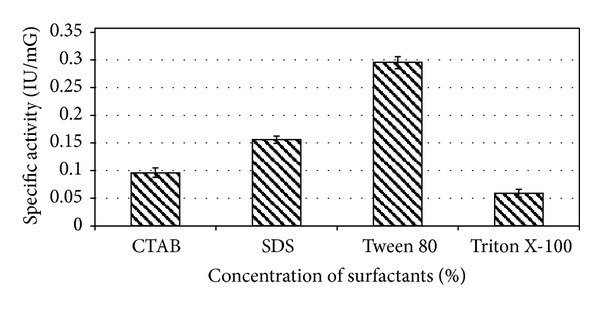
Effect of surfactants on specific ADI activity by *E. faecium* sp. GR7. Results are expressed as the mean ± S.D (*n* = 3), *P*-value <0.05.

Among varying concentrations of metal salts, that is, MnSO_4_, CuSO_4_, and ZnSO_4_, *E. faecium* sp. GR7 showed highest ADI activity of 0.186 ± 0.010 IU/mG at the concentration of 0.6 mM (Figure 10). ADI specific activity was also enhanced by NaCl and K_2_HPO_4_ salts (Figure 11). Among surface active agents, production media showed highest specific ADI activity (0.318 ± 0.012 IU/mG) when supplemented with 0.1% Tween 80 (Figure 12).

A new medium including the best source of carbon, nitrogen, inducer, salts, and surfactants for ADI production was improved, and bacterium *E. faecium* sp. GR7 was grown in this new medium under optimized process parameters and culture conditions lactose 20 g/L; tryptone 20 g/L; arginine 15 mM; MnSO_4_ 0.6 mM; NaCl 1.0 g/L; K_2_HPO_4_ 3 g/L; Tween 80 1%; pH-6.0 at 30°C for 24 h under aerobic conditions. The specific ADI activity by *E. faecium* sp.GR7 was enhanced to 4 folds (0.732 ± 0.006 IU/mG) as compared to TGYE basal media (0.182 ± 0.001 IU/mG).

### 3.7. Central Composite Design

A new medium was formed by selecting most influencing variables from independent experiments, which was further optimized using RSM to enhance enzyme production. In statistical tool CCD, each selected variable, that is, lactose, tryptone, and arginine while keeping MnSO_4_, NaCl, K_2_HPO_4_, Tween 80, and pH-6 at 30°C for 24 h under aerobic conditions as constant factors was studied at five different levels along with other variables, and therefore, the interaction among the variables at their different levels could be studied. When grown in unoptimized TGYE broth, *E. faecium* sp. GR7 showed 0.182 IU/mG of specific ADI activity with a growth of 0.823 at *A*_600 nm_  after 24 h of incubation at 30°C. In order to enhance specific ADI activity and studying growth, experiments were designed to optimize media constituents.

Results obtained were fed into Design-Expert software and analyzed using analysis of variance (ANOVA) as appropriate to the experimental design used. Based on the CCD, the experimental levels of specific ADI activity under each set of condition were determined and compared with the corresponding predicted levels suggested by Design-Expert 8.0.2 (Table 2). The maximum experimental value for ADI specific activity was 4.27 IU/mG (the average of triplicates), while the value of predicted response is 4.29 IU/mG. Approximately 99% of validity was achieved, indicating the model exerted an adequate prediction on the enzyme activity. The close correlation between the experimental and predicted data indicates the appropriateness of the experimental design. The quality of the model can also be checked using various criteria. The calculated regression equation for the optimization of media constituents assessed the specific activity (*Y*) and growth (*G*) as a function of these variables. Multiple regression analysis of the experimental data was carried out, and statistical equation was generated which gives ADI enzyme activity and growth according to (1) and (2), respectively, as follows:

(1) Y=0.8349−0.6999∗A+0.2918∗B+0.5441∗C−1.1857∗A∗B+0.2609∗A∗C−0.7642∗B∗C+0.5288∗A2+0.1475∗B2+0.1248∗C2,

(2) G=1.2325+0.5127∗A+0.3384∗B−0.0850∗C+0.3116∗A∗B+0.0138∗A∗C+0.0386∗B∗C−0.0965∗A2−0.2595∗B2−0.3012∗C2,

where *Y* represents specific enzyme activity, *G* represents growth, and *A*, *B*, *C* are coded values of tryptone, lactose, and arginine, respectively. Tables 5 and 6 show ANOVA results for the RSM quadratic model for *Y* and *G* are responses, respectively. According to the present model *A*, *C*, *AB*, *BC*, *A* ^2^ are significant model terms for *Y* response, and *A*, *B*, *AB*, *B* ^2^, *C* ^2^ are significant model terms for *G* response, respectively.

Quadratic model was found to be the “best fit model” for the specific enzyme activity also for growth with the highest *F*-value when compared to other models (Table 3). ANOVA for ADI specific activity (*Y*, IU/mG) indicated the “*P*-value” to be 0.0018, which implies the model to be significant. The “lack of fit *F*-value” of 99.43 with *P*-value <0.0001 implies that the lack of fit is significant. There is only a 0.01% chance that a “lack of fit *F*-value” this large could occur due to noise. ANOVA indicated the *R*_2_ value of 0.8745 for response *Y*. This ensures a good correlation between observed and expected values of the quadratic model and indicates that this model could explain 87% response variability. The adequate precision which measures the signal-to-noise ratio of 9.227 indicates an adequate signal.

Quadratic model is also found to be the “best fit model” for the growth at *A*_600_ with the highest *F*-value when compared to other models (Table 4). ANOVA for ADI growth (*G*, *A*_600_) indicated the “*P*-value” to be 0.0004, which implies the model to be significant. The “lack of fit *P*-value” of <0.0002 implies the lack of fit is significant. There is only a 0.02% chance that a “lack of fit *F*-value” this large could occur due to noise. A 91% response variability showed good correlation between observed and expected values of the quadratic model as ANOVA indicated the *R* ^2^ value of 0.9099 for response *Y*. The adequate precision which measures the signal-to-noise ratio of 9.58 indicates an adequate signal.

**Figure 13:**
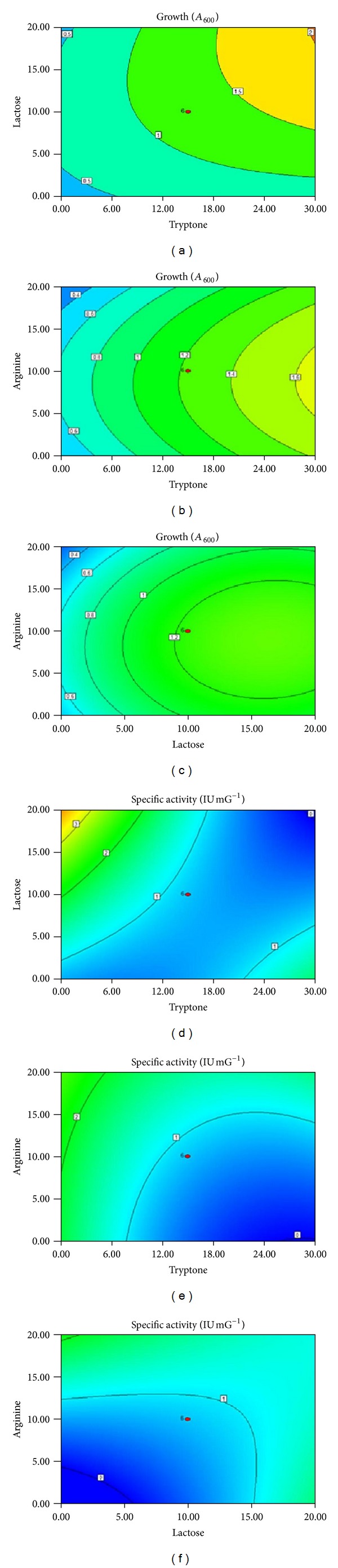
2D contour plot showing effects and interaction of (a) lactose (g/L) and tryptone (g/L) (b) arginine (g/L) and tryptone (g/L) (c) arginine (g/L) and lactose (g/L) on growth at *A*_600_ of *E. faecium* isolate GR7 and (d) lactose (g/L) and tryptone (g/L) (e) arginine (g/L) and tryptone (g/L) (f) arginine (g/L) and lactose (g/L) on specific ADI activity of *E. faecium* sp. GR7.

*E. faecium *sp. GR7 shows very poor specific ADI activity (0.182 ± 0.001 IU/mG) in unoptimized TGYE broth, which was enhanced to 0.732 ± 0.006 IU/mG in new media and finally raised to 15 folds (4.27 IU/mG) in RSM optimized media (run 16) as shown in Table 2. The 2D contour plots based on the interactions between these variables show correlation between increase in specific ADI activity versus concentration of each variable that reaches the optimum level of enzyme activity and growth at concentration (g/L) lactose 10; tryptone 15; arginine 20 beyond which a decline was observed (Figures 13(d), 13(e), and 13(f)). 2D contour plots were obtained by plotting any two variables on *x* and *y*-axis while keeping the other third variable at their central value, that is, lactose 10 g/L, 15 g/L tryptone, and arginine 10 g/L. CCD employed has revealed that tryptone at concentration of 15 g/L was optimal for maximum enzyme activity. This nitrogen source plays important role in ADI specific activity and biomass production, as growth increases with the concomitant increase in tryptone concentration (Figures 13(a), 13(b), 13(d), and 13(e)). Results indicate a central value of lactose as 10 g/L for optimal enzyme production. In present study, similar effect was observed in case of lactose, as the concentration of lactose increases their is an increase in biomass production (Figures 13(a) and 13(c)). But it represses ADI enzyme activity as its concentration increases beyond 10 g/L (Figures 13(d) and 13(f)). Concentration of arginine in the culture medium also has profound effect on specific activity of arginine deiminase (Figures 13(e) to 13(f)), but it is not required for bacterial growth.

**Figure 14:**
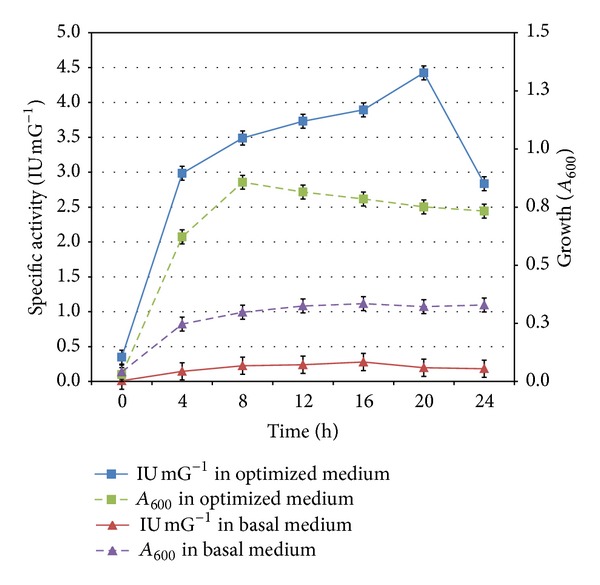
Time course of specific ADI activity and growth of *E. faecium* sp. GR7 in optimized and basal (TGYE) medium. Results are expressed as the mean ± S.D (*n* = 3), *P*-value <0.05.

*E. faecium* sp. GR7 when grown in media containing different nutrients resulted in different growth profiles. The growth (*A*_600_) was mostly influenced by an increase in the concentration of tryptone and lactose (Figure 13(a)). Presence of arginine in the culture medium has a least contribution towards growth of *E. faecium* sp. GR7 (Figures 13(b) and 13(c)), although it is important for inducing ADI activity (Figures 13(e) and 13(f)). Based on the results obtained, optimized medium designed with the components (g/L) tryptone 15; lactose 10; arginine 20; NaCl 1.0; K_2_HPO_4_ 3.0; MnSO_4_ 0.6 mM; Tween 80 1%; pH-6.0 at 30°C for 24 h under aerobic conditions.  Figure 14 illustrates comparative growth profiles of the *E. faecalis* strain in basal (TGYE) and optimal media. Biomass concentration of the bacteria cultivated in optimal medium in batch culture conditions reached 0.751 at *A*_600_ (12.1 mg/L) after 20 h of incubation with concomitant production of 4.27 IU mG^−1 ^IU/mG specific ADI activity. This being higher in comparison with bacteria in TGYE medium with 0.32 at *A*_600_ (5.17 mg/L) biomass after 20 h of incubation with specific ADI activity of 0.20 IU/mG. Though the biomass yield is lesser in optimized medium but higher specific ADI activity is having more significance from industrial perspective.

## 4. Discussion

ADI pathway of bacteria provides number of functions such as sole energy provider (ATP) for the growth of a variety of microorganisms in various environmental conditions, especially arginine as main catabolite for nonfermenting bacteria, allowing anaerobic growth of strict aerobic bacteria, growth of fermentative bacteria when sugars are not available or at low sugar concentration, to supply carbamoyl phosphate for biosynthesis of citrulline or pyrimidines and to protect bacteria against acid or starvation environmental stress conditions [12, 30]. Therefore, expression of ADI pathway is widely reported among prokaryotic organisms including prominent bacterial species such as *Clostridium* [19], *Enterococcus* [20], *Halobacterium* [31], *Lactobacillus* [21], *Lactococcus* [6], *Leuconostoc* [7], *Mycoplasma* [22], *Pseudomonas* [25], *Streptococcus* [26], and *Weissella* [27]. Arginine catabolism by the ADI pathway has been explored in several industrial lactic acid bacterial species, mainly regarding their genetic and physiological aspect and enzymology of the ADI pathway. The increase in acid resistance of homofermentative and heterofermentative LAB may be due to the restoration of the optimum intercellular pH through arginine utilization and ammonia production [12]. Few reports emphasizing the role of the media constituents for enhancing ADI activity in bacterial species are available [23, 24, 27]. More knowledge about the influence of media components on ADI production, especially in GRAS LAB, is desired, which populate different food environments. In the present work, production of arginine deiminase in *E. faecium* sp. GR7 was enhanced to 4 folds in fermentation media (EAPM) using independent experiments which was further raised by 15 folds in RSM optimized media supplemented with 15 mM arginine in comparison to basal medium. Specific ADI activities reported in GRAS LAB range from 0.10 to 2.80 IU/mG (Table 7). Previous reports on *Lactobacillus buchneri* CUC-3 [32], *Lactobacillus buchneri* NCDO_110_ [29], *Lactobacillus sanfranciscensis* CB1 [12], *Streptococcus lactis* [9], *Weissella confusa* GR7 [27], *Weissella koreensis *MSI-3 [10], and *Weissella koreensis* MSI-14 [10] have suggested the role of arginine in enhancing ADI activity in various LAB strains. Although, arginine contributes little to the growth of *E. faecium *sp. GR7 but its involvement in induction of ADI activity is reported in the present study. The nature as well as concentration of the sugars was found to affect ADI production in case of lactose metabolizing *E. faecium* sp. GR7. Other sugars such as glucose, galactose, sucrose, maltose, and fructose did not suppress induction of ADI which was reported earlier in other LAB strains such as *Lactobacillus buchneri* NCDO_110_ (glucose) [29], *Lactobacillus sake* (above 0.6 mM glucose) [31], *Lactobacillus sanfranciscensis* CB1 (above 54 mM glucose) [12], and *Streptococcus lactis* (glucose, lactose) [9]. Fructose was known to suppress ADI activity in *Leuconostoc oenos, Streptococcus mitis, *and* Weissella confusa* GR7 [27, 33, 34]. Tryptone showed highest ADI production in *E. faecium* sp. GR7 at (10 g/L) which was approximately 11.57 and 8.05 times higher than yeast extract, and peptone respectively, as reported earlier in *E. faecalis* NJ402 [21]. ADI pathways also exhibit salt dependence as MnSO_4_, NaCl, and K_2_HPO_4_ are reported to magnify their activity [35].

Conventional processes of optimization are usually time consuming and expensive. The single factor optimization cannot explain the actual interactions of the parameters of the experimental data because the interaction between different factors is overlooked, leading to a misinterpretation of the results. In this study, optimization of multiple factors yielded more accurate results from which one could choose the correct value of each response factor individually with a high certainty. Fermentation media (EAPM) optimized using single factor based independent experiments resulted in 4-fold increase in ADI specific activity; however, a 15-fold increase was observed in RSM optimized medium when compared with basal medium. The optimized media components could be selected as the best condition for further studies on the development of a cost effective and viable industrial fermentation process. Thus, this work demonstrated the advantage of statistical media optimization methods for enzyme production with a minimum number of experimental trials, simultaneously with a reduction in laborious and time-consuming media composition analysis strategies.

## 5. Conclusion

A new strain of an *Enterococcus* was found to be a potential producer of ADI enzyme. In this study, result of bioprocess was optimized for future scaleup of ADI production process in *E. faecium* sp. GR7. A statistical strategy of CCD was used successfully to find out optimum values of significant response factors, which resulted in 15-fold increase in ADI production in RSM optimized media over basal media in *E. faecium* sp. GR7. It is interesting to report a novel lactic acid bacterial strain; that is, *E. faecium* having arginine metabolizing capacity can be ideally explored for production as a class of therapeutic enzyme against arginine auxotrophic cancers at low cost where the industrial needs are satisfied. The progress in the ADI research will undoubtedly facilitate the use of amino acid depriving enzymes in an innovative strategy in therapy of specific auxotrophic tumors and have profound influence on human health.

## Figures and Tables

**Table 1:** Variables and their levels for arginine deiminase production by *E. faecium* sp. GR7.

Factor (g/L)	Low level star point (−*α*)	Low level factorial (−1)	Central point (0)	High level factorial (+1)	High level star point (+*α*)
A-tryptone	−0.23	0	15	30	40.23
B-lactose	−6.82	0	10	20	26.87
C-arginine	−6.82	0	10	20	26.817

**Table 2:** Experimental design and results of central composite design for ADI specific activity and growth from *E. faecium* sp. GR7.

Run	Tryptone	Lactose	Arginine	Growth (*A*_600_)		Specific activity (IU/mG)	
	(g/L)	(g/L)	(g/L)	Predicted value	Experimental value	Predicted value	Experimental value
1	0	0	0	1.771	1.156	0.001	0.839
2	0	−*α*	0	1.822	0.103	1.153	0.172
3	0	0	+*α*	0.328	0.185	2.103	0.888
4	−1	−1	−1	1.706	0.002	0.083	0.0002
5	+1	+1	+1	0.029	1.607	3.332	0.643
6	−1	+1	−1	1.232	0.054	0.835	4.169
7	−1	−1	+1	1.232	0.022	0.835	2.406
8	+1	+1	−1	−0.102	1.377	1.906	0.251
9	0	0	0	1.232	1.249	0.835	0.839
10	+1	−1	−1	0.237	0.259	2.103	0.295
11	0	0	0	0.548	1.156	0.261	0.839
12	0	0	0	1.232	1.249	0.835	1.056
13	0	0	0	1.232	1.26	0.835	0.779
14	+*α*	0	0	0.149	2.26	0.761	0.492
15	0	+*α*	0	1.232	1.276	1.743	1.272
**16**	**+1**	**0**	**+1**	**0.322**	**0.154**	**4.295**	**4.274**
17	+1	−1	+1	0.029	0.028	3.399	4.034
18	0	0	0	0.173	1.26	−0.189	0.839
19	−*α*	0	0	1.068	0.041	1.743	3.109
20	0	0	−*α*	0.097	0.958	3.508	0.428

**Table 3:** Model fit summary for the growth.

Source	Sum of squares	Degree of freedom	Mean square	*F* value	*P* value	Prob > *F *
Mean versus total	12.29	1	12.29			
Linear versus mean	5.25	3	1.75	7.57	0.0023	
2FI versus linear	0.79	3	0.26	1.18	0.3567	
Quadratic versus 2FI	2.11	3	0.70	8.70	0.0039	Suggested
Cubic versus quadratic	0.43	4	0.11	1.70	0.2674	Aliased
Residual	0.38	6	0.06			

Total	21.24	20	1.06			

**Table 4:** Model fit summary for ADI specific activity.

Source	Sum of squares	Degree of freedom	Mean square	*F* value	*P* value	Prob > *F *
Mean versus total	38.20	1	38.20			
Linear versus mean	11.90	3	3.97	2.50	0.0963	
2FI versus linear	16.47	3	5.49	8.02	0.0028	Suggested
Quadratic versus 2FI	4.22	3	1.41	3.01	0.0811	Suggested
Cubic versus quadratic	1.81	4	0.45	0.95	0.4966	Aliased
Residual	2.86	6	0.48			

Total	75.46	20	3.77			

**Table 5:** Regression analysis (ANOVA) for the growth.

Source	Sum of squares	Degree of freedom	Mean squares	*F* value	*P* value	Prob > *F *
Model	8.150	9	0.960	11.228	0.0004	Significant
A-tryptone	3.591	1	3.591	44.527	<0.0001	
B-lactose	1.564	1	1.564	19.395	0.0013	
C-arginine	0.099	1	0.099	1.224	0.2945	
AB	0.777	1	0.777	9.633	0.0112	
AC	0.002	1	0.002	0.019	0.8928	
BC	0.012	1	0.012	0.148	0.7085	
A^*∧*^2	0.134	1	0.134	1.664	0.2260	
B^*∧*^2	0.970	1	0.970	12.034	0.0060	
C^*∧*^2	1.308	1	1.308	16.241	0.0024	
Residual	0.806	10	0.081			
Lack of fit	0.793	5	0.159	60.763	<0.0002	Significant
Pure error	0.013	5	0.003			
Cor total	8.956	9				

Coefficient of determination (*R* ^2^) = 0.909.

**Table 6:** Regression analysis (ANOVA) for ADI specific activity.

Source	Sum of squares	Degree of freedom	Mean squares	*F* value	*P* value	Prob > *F *
Model	32.586	9	3.621	7.749	0.0018	Significant
A-tryptone	6.692	1	6.692	14.321	0.0036	
B-lactose	1.163	1	1.163	2.489	0.1457	
C-arginine	4.043	1	4.043	8.653	0.0147	
AB	11.249	1	11.249	24.074	0.0006	
AC	0.545	1	0.545	1.166	0.3056	
BC	4.673	1	4.673	10.001	0.0101	
A^*∧*^2	4.030	1	4.030	8.625	0.0149	
B^*∧*^2	0.314	1	0.314	0.671	0.4317	
C^*∧*^2	0.225	1	0.225	0.481	0.5038	
Residual	4.673	10	0.467			
Lack of fit	4.626	5	0.925	99.432	<0.0001	Significant
Pure error	0.047	5	0.009			
Cor total	37.258	19				

Coefficient of determination (*R* ^2^) = 0.874.

**Table 7:** Specific ADI activity in different microorganisms.

Native microorganism	Specific ADI activity (IU/mG)	Reference
*Clostridium sporogenes *	2.0	[19]
***Enterococcus faecalis* NJ402 **	0.238	[20]
*Halobacterium salinarum *	0.33 nKat/mg	[31]
***Lactobacillus sake***	1.5 U/mL	[21]
***Lactobacillus buchneri* CUC-3**	1.01	[7]
***L. sanfranciscensis *CB1**	16.20 (from 10 times concentrated suspension)	[12]
***Lactococcus lactis *ssp*. lactis *ATCC 7962 **	2.16	[6]
***Leuconostoc oenos* OENO**	0.10	[7]
*Mycoplasma arginini *ATCC 23243	1.91	[22]
*Mycoplasma arginini *G-230	1.37	
*Mycoplasma arginini *strain *leonis *	0.78	
*Mycoplasma arthritidis *	1.2	
*Mycoplasma fermentans *	0.28	
*Mycoplasma gallinarum *	1.3	
*Mycoplasma hominis *ATCC 14027	2.0	
*Mycoplasma hominis *	2.9	
*Pseudomonas plecoglossicida *CGMCC2039		
Wild type strain		
Before media optimization	0.96 U/mL	[23]
After media optimization	1.6 U/mL	
Mutant strain		
At pH 6.0	16.7	[24]
At pH 6.5	21.7	
*Pseudomonas putida *	1.52	[25]
***Streptococcus faecalis *13398**	1.60	[26]
***Streptococcus faecium* A-2**	0.11	[26]
***Streptococcus lactis ML3***	0.28	[9]
***Weissella confusa * GR7 **		[27]
Before media optimization	0.27	
After media optimization	2.808	
***Enterococcus faecium *GR7 **		Present research work
Before media optimization	0.182	
After media optimization	4.27	
